# Behavior of Hypertrophied Right Ventricle during the Development of Left Ventricular Failure Due to Myocardial Infarction

**DOI:** 10.3390/ijms25052610

**Published:** 2024-02-23

**Authors:** Naranjan S. Dhalla, Karina Oliveira Mota, Carla Maria Lins de Vasconcelos, Adriana Adameova

**Affiliations:** 1 Institute of Cardiovascular Sciences, St. Boniface Hospital Albrechtsen Research Centre, Winnipeg, MB R2H 2A6, Canada; 2 Department of Physiology and Pathophysiology, Rady Faculty of Health Sciences, College of Medicine, University of Manitoba, Winnipeg, MB R3E 0J9, Canada; 3 Heart Biophysics Laboratory, Department of Physiology, Center for Biological and Health Sciences, Federal University of Sergipe, São Cristóvão 49100-000, Brazil; karynamota@academico.ufs.br (K.O.M.); carlamlv@academico.ufs.br (C.M.L.d.V.); 4 Department of Pharmacology and Toxicology, Faculty of Pharmacy, Institute for Heart Research, Slovak Academy of Sciences, 8H103 Bratislava, Slovakia; adriana.duris.adameova@uniba.sk

**Keywords:** cardiac hypertrophy, β_1_-adrenoreceptors, adenylyl cyclase, protein kinase C, phospholipase C, protein kinase A, G-proteins, sarcoplasmic reticulum Ca^2+^-transport, myofibrillar Ca^2+^-ATPase

## Abstract

In order to determine the behavior of the right ventricle, we have reviewed the existing literature in the area of cardiac remodeling, signal transduction pathways, subcellular mechanisms, β-adrenoreceptor-adenylyl cyclase system and myocardial catecholamine content during the development of left ventricular failure due to myocardial infarction. The right ventricle exhibited adaptive cardiac hypertrophy due to increases in different signal transduction pathways involving the activation of protein kinase C, phospholipase C and protein kinase A systems by elevated levels of vasoactive hormones such as catecholamines and angiotensin II in the circulation at early and moderate stages of heart failure. An increase in the sarcoplasmic reticulum Ca^2+^ transport without any changes in myofibrillar Ca^2+^-stimulated ATPase was observed in the right ventricle at early and moderate stages of heart failure. On the other hand, the right ventricle showed maladaptive cardiac hypertrophy at the severe stages of heart failure due to myocardial infarction. The upregulation and downregulation of β-adrenoreceptor-mediated signal transduction pathways were observed in the right ventricle at moderate and late stages of heart failure, respectively. The catalytic activity of adenylate cyclase, as well as the regulation of this enzyme by G_s_ proteins, were seen to be augmented in the hypertrophied right ventricle at early, moderate and severe stages of heart failure. Furthermore, catecholamine stores and catecholamine uptake in the right ventricle were also affected as a consequence of changes in the sympathetic nervous system at different stages of heart failure. It is suggested that the hypertrophied right ventricle may serve as a compensatory mechanism to the left ventricle during the development of early and moderate stages of heart failure.

## 1. Introduction

It is now well recognized that heart failure is a complex cardiovascular disorder which is invariably preceded by cardiac hypertrophy. Numerous pathological conditions such as hypertension, diabetes, obesity, aging, atherosclerosis, myocardial infarction, valvular heart disease and different types of cardiomyopathies are known to cause cardiac hypertrophy and heart failure upon increasing ventricular tension as a consequence of increased pre-load or after-load, as well as elevated levels of several vasoactive hormones, including catecholamines and angiotensin II [1,2,3,4,5,6]. Although the muscle mass is increased in both cardiac hypertrophy and heart failure stages, the ventricular function and cardiac output are depressed in heart failure, whereas ventricular function in the hypertrophied (non-failing) heart is either unchanged or upregulated. Likewise, the upregulation and downregulation of cardiac metabolism, Ca^2+^ handling by cardiomyocytes, diverse signal transduction pathways and adrenoreceptor-associated responses have been observed at early stages of cardiac hypertrophy and at heart failure stages [1,3,6,7,8,9,10]. It should be mentioned that cardiac hypertrophy at early stages is compensatory or adaptive in nature and is termed as physiological hypertrophy, whereas at later stages, it is termed as pathological hypertrophy, which is considered a risk factor for the development of heart failure [11]. Furthermore, these characteristics of cardiac hypertrophy and heart failure due to increased workload (pressure overload and volume overload) are attributed on the basis of observations of the behavior of the left ventricle during different pathological conditions. Thus, physiological hypertrophy of the left ventricle is considered a compensatory mechanism for maintaining left ventricle function during the development of heart failure.

While extensive research work has been carried out to understand the cellular and molecular mechanisms responsible for the occurrence of left ventricular hypertrophy and failure, relatively very little work has been done to understand behavior of the right ventricle during the development of heart failure. Sustained pressure overload on the right ventricle induced by pulmonary artery occlusion in dogs was observed to increase the muscle mass, fiber diameter and transmural blood flow in the right ventricle [12]. The occurrence of acute right ventricle hypertrophy in cats by pulmonary artery banding was found to be associated with increased Ca^2+^ uptake by the sarcoplasmic reticulum, whereas chronic hypertrophy of the right ventricle was associated with depression of the sarcoplasmic reticulum Ca^2+^ uptake and protein yield [13]. It should be noted that measurement of the hemodynamic status as well as electrophysiological and contractile parameters in a cat model of pulmonary artery constriction has revealed that chronic right ventricular hypertrophy, unlike acute cardiac hypertrophy, is associated with cardiac failure [13,14]. Furthermore, the observed alterations in Ca^2+^ uptake activity in acute and chronic right ventricular hypertrophy due to pulmonary hypertension are similar to the enhanced Ca^2+^ transport activity in left ventricular hypertrophy in rats [15], as well as augmented and depressed Ca^2+^ pump activities in pig left ventricular physiological and pathological hypertrophy due to pressure overload, respectively [16]. Differential alterations in behavior of the right ventricle with respect to β-adrenoreceptor-associated and protein kinase C- associated signal transduction mechanisms in adaptive cardiac hypertrophy and failing hearts due to volume overload have also been observed [17,18,19]. It is pointed out that the most common cause of right ventricular failure has been shown to be left ventricular failure in humans [20,21]. It is also noteworthy that lower Ca^2+^ transport activity in the right ventricular sarcoplasmic reticulum has been shown to account for the lower ability of the right ventricle to generate contractile force in comparison to the left ventricle [22]. Thus, behavior of the right ventricle seems to be different from that of the left ventricle under both physiological and pathological conditions.

In view of the fact that heart failure due to myocardial infarction is the most prevalent cardiovascular disorder, this article is aimed to review the state-of-the-art information regarding the behavior of the right ventricle during the development of cardiac hypertrophy and heart failure. It is pointed out that on the basis of hemodynamic, morphometric and biochemical analysis, a rat model of myocardial infarction with a large infarct size (>30%) in the left ventricle is considered most suited for studying alterations in the right ventricle during the development of heart failure [23,24,25,26,27,28,29]. Since this experimental rat model has been demonstrated to exhibit early, moderate and severe stages of heart failure at 4, 8 and 16 weeks of inducing myocardial infarction, respectively [29], it is intended to highlight changes in the right ventricle at different stages of heart failure. Particularly, alterations in the right ventricle will be described with respect to cardiac hypertrophy and associated signal transduction mechanisms, as well as cardiac function and associated sarcoplasmic reticulum and myofibrillar activities. Because of the derangement of β-adrenoreceptor-associated mechanisms in heart failure [10,30], the existing information for changes in β-adrenoreceptors, adenylyl cyclase and G proteins in the right ventricle will be reviewed. In addition, the role of sympathetic activation in the development of alterations in the right ventricle will be discussed.

## 2. Development of Right Ventricular Hypertrophy

The data in Table 1 indicate that there was a progressive increase in the muscle mass of the right ventricle in rats at 4, 8, 16 and 24 weeks after the induction of myocardial infarction. This alteration was associated with an increase in the left ventricular muscle mass at 8 to 24 weeks, except at 4 weeks, due to myocardial infarction; no changes in scar weight were apparent during this period. However, on the basis of abdominal fluid accumulation (ascites) and changes in left ventricular function (Table 1) and other hemodynamic data, the infarcted animals at 4, 8, 16 and 24 weeks were considered to be at early, moderate, severe and late stages of heart failure, respectively [29,31,32]. Progressive left ventricular remodeling and cardiac hypertrophy, as well as alterations in hemodynamic status of rats with large infarct size, have also been observed by other investigators [23,24,25,26,27,28]. It should be pointed out that myocyte hypertrophy showed an increase in both diameter and length in the left ventricle, whereas myocyte hypertrophy in right ventricle exhibited an increase in diameter only [33].

Since different signal transduction mechanisms, including protein kinase C, mitogen-activated protein kinase, protein kinase A and phospholipase C, are involved in the hypertrophic process of the left ventricle [6,34,35], it likely that right ventricle hypertrophy may be a consequence of the activation of such signaling pathways. In this regard, it may be noted from data in Table 2 that right ventricle hypertrophy is associated with increased activities of both Ca^2+^-dependent and Ca^2+^-independent protein kinase activities at 2 weeks (pre-failure), 4 weeks (early failure) and 8 weeks (moderate failure) after the induction of myocardial infarction [34]. Furthermore, different protein kinase C isozymes such as PKC-α, PKC-β and PKC-ℇ, unlike PKC-ꞔ, were activated in the right ventricle at the moderate stage (8 weeks) of heart failure following myocardial infarction (Table 3). However, no changes in protein kinase A content or activity were observed in the hypertrophied right ventricle after 8 weeks of myocardial infarction (Table 3) [34]. No alterations in phospholipase C activity, nor in maximal activation or substrate affinity (1/K_m_) for this enzyme, were seen in the hypertrophied right ventricle at 8 weeks (moderate stage of heart failure) after the induction of myocardial infarction (Table 4) [35]. On the other hand, 16 weeks (severe stage of heart failure) after the induction of myocardial infarction, phospholipase C activity and substrate affinity (1/K_m_) were depressed without any changes in maximal activation of the enzyme (Table 4) [35]. These results show that the PKC–inositol-3-phosphate signaling pathway may be directly involved in inducing cardiac hypertrophy in the right ventricle for maintaining its function upon inducing myocardial infarction, whereas depressed phospholipase C activity as well as its substrate affinity may produce a decrease in the ability of the right ventricle to generate contractile force and limit the hypertrophic response as a consequence of a decrease in the formation of inositol-3-phosphate and subsequent Ca^2+^ release from the intracellular Ca^2+^ stores [35]. Although the activities of protein kinase A and phospholipase C in the hypertrophied right ventricle were not altered upon the induction of myocardial infarction, these pathways may be indirectly involved in the hypertrophic processes through elevated levels of norepinephrine during early periods [36].

## 3. Subcellular Remodeling in the Hypertrophied Right Ventricle

By virtue of their ability to regulate the intracellular concentration of Ca^2+^ in cardiomyocytes, remodeling of both the sarcolemma and the sarcoplasmic reticulum is known to determine the status of ventricular function [3]. While the density of sarcolemmal Ca^2+^ channels was depressed in both moderate and severe left ventricular dysfunction, no change in this parameter was observed in the right ventricle at any stage of heart failure [29]. Both sarcolemmal Na^+^–Ca^2+^ exchange and Na^+^–K^+^ pump activities were depressed in the failing left ventricle, but these parameters were not determined in the right ventricle of the infarcted heart [37]. On the other hand, sarcoplasmic reticulum Ca^2+^ uptake activity, as well as maximal Ca^2+^ uptake (V_max_), in the right ventricle from 4 weeks and 8 weeks of myocardial infarction were increased without any change in the affinity for Ca^2+^ (Table 5) [37]. Although Ca^2+^ uptake values in the right ventricle after 16 weeks of myocardial infarction were lightly (12 to 16%) depressed, these were not significant (Table 5). It should also be mentioned that Ca^2+^-stimulated ATPase activity in the sarcoplasmic reticulum in right ventricles infarcted for 4 and 8 weeks was increased [37]. In contrast, Ca^2+^ uptake activities in the sarcoplasmic reticulum from the left ventricle 4, 8 and 16 weeks after myocardial infarction induction were markedly decreased [37]. It should be mentioned that sarcoplasmic reticulum Ca^2+^ uptake was augmented in right ventricular hypertrophy and depressed in the failing myocardium upon banding of the pulmonary artery [13]. Physiological cardiac hypertrophy due to swimming [38], as well as mild or early cardiac hypertrophy due to pressure overload or upon administration of catecholamines, have also been shown to be associated with increased sarcoplasmic reticulum Ca^2+^ uptake activities [15,16,39].

It is noteworthy to point out that the right ventricular function has been reported to be augmented during early periods [40] but become depressed over time upon the induction of myocardial infarction [27,28,41]. The hyperfunction of the hypertrophied right ventricle during early periods (4 to 8 weeks) of inducing myocardial infarction may be related to augmented Ca^2+^ uptake in the sarcoplasmic reticulum, as indicated in Table 5. Since myofibrillar remodeling has been reported to participate in determining cardiac function [3], it is possible that changes in myofibrillar activities may also be associated with changes in right ventricular function due to myocardial infarction. From the data in Table 6, it can be seen that the activity of myofibrillar Ca^2+^-stimulated ATPase, which is known to determine cardiac contractile force development, was not altered in the right ventricle at 4 weeks or 8 weeks after myocardial infarction (Table 6) [42]. Because the activity of myofibrillar Ca^2+^-stimulated ATPase in the right ventricle was significantly depressed in the heart 16 weeks after induced infarction (Table 6), it appears that this defect may be associated with delayed dysfunction of the right ventricle. Furthermore, the distribution of myosin-α and myosin-β isozymes, as well as changes in their corresponding mRNA expression in the right ventricle 8 weeks after infarction (Table 6), may be a reflection of myocardial hypertrophy.

Variations with respect to cardiac actomyosin ATPase and myosin isozymes have been observed in the right and left ventricles during the development of myocardial infarction [43], and changes in both α-myosin and β-myosin in both ventricles due to infarction were prevented by treatment with a Ca^2+^ antagonist, amlodipine [44]. Although alterations in myofibrillar ATPase as well as changes in myosin isozymes in the left ventricle due to myocardial infarction were modified by treatment with angiotensin-converting enzyme (enalapril) as well as an angiotensin antagonist (losartan), the effects of these interventions were not determined in the right ventricle [45]. Nonetheless, extensive research work regarding alterations in the activities of both myofibrils and the sarcoplasmic reticulum needs to be carried out for establishing the subcellular basis of right ventricle hypertrophy and cardiac dysfunction during the development of heart failure due to myocardial infarction.

## 4. β-Adrenoreceptor Mediated Signal Transduction in Right Ventricle

Earlier, we reviewed the status of β_1_-adrenoreceptor signal transduction pathway in cardiac hypertrophy and heart failure due to pressure overload and volume overload and reported upregulation in adaptive (physiological) hypertrophy and downregulation of these mechanisms in pathological (maladaptive) hypertrophy or failing hearts [8,10]. We have also carried out studies showing the downregulation of adrenoreceptor mechanisms in the failing left ventricle both at moderate and late stages of heart failure subsequent to myocardial infarction [31,32,46]. In this article, we have reviewed the data regarding the status of different components of β-adrenoreceptor signal pathway in the right ventricle at 4, 8, 16 and 24 weeks of heart failure due to myocardial infarction. The results in Table 7 show changes in the intracellular Ca^2+^ in myocytes obtained from the right ventricle at 4, 8, 16 and 24 weeks of heart failure due to myocardial infarction. The results in Table 7 show changes in intracellular Ca^2+^ in myocytes obtained from the right ventricle at 8 weeks (moderate stage) and 24 weeks (late stage) of heart failure as a consequence of myocardial infarction. Although basal [Ca^2+^]_i_ levels in cardiomyocytes at 8 weeks were not different from those at 24 weeks, both KCl-induced and isoproterenol-induced increases in [Ca^2+^]_i_ were augmented at 8 weeks and depressed at 24 weeks. Furthermore, the β-adrenoreceptor density was unaltered at 8 weeks and decreased at 24 weeks, without any changes in the affinity of these receptors for the binding radioligand (Table 8). In addition, adenylyl cyclase activities in the right ventricle membranes in the absence (basal) and presence of isoproterenol were increased at 8 weeks and depressed at 24 weeks after inducing myocardial infarction (Table 8). These observations suggest that the upregulation of β-adrenoreceptor-induced [Ca^2+^]_i_ increase in the hypertrophied right ventricle myocyte 8 weeks after myocardial infarction was due to increased activity as well as activation of adenylyl cyclase. On the other hand, depressed responses of [Ca^2+^]_i_ increase were associated with decreases in both β-adrenoreceptor density and the activation of adenylyl cyclase by isoproterenol.

The status of β_1_-adrenoreceptors and adenylyl cyclase was also determined in the right ventricle membranes from hearts at early (4 weeks), moderate (8 weeks) and severe (16 weeks) stages of heart failure due to myocardial infarction, and the results are shown in Table 9 [46]. No changes in the densities or the affinities of either β_1_- or β_2_-adrenoreceptors in the hypertrophied ventricle were evident at different stages of heart failure. However, the adenylyl cyclase activity in the presence of isoproterenol at 8 weeks and 16 weeks was augmented but was depressed at 4 weeks of myocardial infarction (Table 9). The adenylyl cyclase activity in the absence (basal) and presence of various agents such as NaF, forskolin and Gpp (NH)p, which are known to stimulate the enzymes at different sites, was also determined in the right ventricle at different stages of heart failure due to myocardial infarction (Table 10) [46]. The adenylyl cyclase activities in the absence or presence of different stimulants at 8 and 16 weeks of myocardial infarction were increased, whereas those at the 4 weeks were depressed with respect to the respective control valves (Table 10). The exact reason for the stage-dependent differences in the absence or presence of different stimulants remains to be investigated. However, elevated levels of cyclic AMP content in the right ventricle upon injecting isoproterenol or forskolin (10 μg/kg) to animals 8 weeks after infarction were also observed in comparison to the control animals [46].

Since both stimulatory (G_s_) and inhibitory (G_i_) G proteins are known to regulate adenylyl cyclase and thus play an important role in regulating cardiac function [30], adenylyl cyclase and ADP ribosylation (determinant of G-protein activity) in the right ventricle were determined in the absence or presence of G-protein stimulants at different stages of induced myocardial infarction (Table 11 and Table 12) [31]. The data in Table 11 show that pertussis toxin (stimulator of G_i_ protein) increased the adenylyl cyclase activity at 4 weeks (early failure stage) but did not affect the adenylyl cyclase activity at 8 or 16 weeks (moderate or severe stages of heart failure) after inducing myocardial infarction. A similar pattern of changes in pertussis toxin-catalyzed ADP ribosylation in the right ventricle was also seen at 4 and 8 or 16 weeks after inducing myocardial infarction (Table 11). In addition, G_iα_ protein content of the right ventricle was markedly higher at 4 weeks, unlike that at 8 or 16 weeks after inducing myocardial infarction (Table 11). On the other hand, the data in Table 12 revealed that cholera toxin (stimulator of G_s_ protein) depressed the adenylyl cyclase activity at 4 weeks stage but stimulated the enzyme at 8 or 16 weeks after inducing myocardial infarction (Table 12) [31]. Likewise, the G_s_-protein activity (as expressed by cholera toxin-catalyzed ADP ribosylation) at 45 KD and 52 KD bands was unaltered at 4 weeks but stimulated at 8 or 16 weeks after the induction of myocardial infarction (Table 12). Furthermore, G_s_-protein content at 45 KD and 52 KD bands was unaltered at 4 weeks and increased at 8 or 16 weeks after inducing myocardial infarction (Table 12). The observations recorded in Table 11 and Table 12 indicate that the adenylyl activities and their responses to various stimulants were dependent upon the stage of myocardial infarction as a consequence of differences in the G-protein activities.

## 5. Mechanisms of Right Ventricular Remodeling Due to Myocardial Infarction

The loss of cardiac tissue due to infarct formation is known to produce depression in cardiac output immediately and activate both the sympathetic nervous system and renin-angiotensin system mainly [10,30]. The activation of these neurohormonal systems results in elevated levels of catecholamines and angiotensin II in the circulation for the maintenance of cardiac function. Although both these vasoactive hormones produce cardiac hypertrophy by increasing ventricular tension, remodeling of the left ventricle has been shown to be different from that of the right ventricle. There is evidence to show that remodeling and failure of the left ventricle is associated with an increase in both pressure overload and volume overload, whereas right ventricular remodeling and dysfunction occur as a consequence of pressure overload only [47,48,49,50]. Accordingly, the behavior of right and left ventricles for remodeling and functional responses to catecholamines and angiotensin II during the development of myocardial infarction may be a consequence of differences in the signal transduction mechanisms for these and other vasoactive hormones. It should be emphasized that cardiac hypertrophy is an adaptive and compensatory mechanism at early stages; however, prolonged exposure of the hypertrophied heart to both catecholamines and angiotensin II results in cardiac dysfunction and heart failure.

In view of the relative paucity of information regarding angiotensin II-mediated signal transduction and other cellular mechanisms in the right ventricle during myocardial infarction, it is considered appropriate to further discuss the role of catecholamine-related mechanisms for understanding the behavior of the right ventricle. Differential alterations in behavior of the right and left ventricles due to myocardial infarction can be explained on the basis of differential changes in catecholamine stores, norepinephrine release, norepinephrine uptake and norepinephrine turnover in both ventricles during the development of myocardial infarction [36,51,52,53]. The data in Table 13 [36] show that plasma levels of norepinephrine and epinephrine at both 4 and 8 weeks, except norepinephrine levels at 4 weeks, were markedly increased after myocardial infarction. In contrast to the left ventricle, which showed no changes in norepinephrine concentration at 4 and 8 weeks [36], norepinephrine concentration in the right ventricle was increased at 8 weeks only (Table 13). Although epinephrine concentration in the right ventricle was increased at both 4 and 8 weeks after the induction of myocardial infarction (Table 13), these changes in the right ventricle were less than those in the left ventricle [36]. The accumulation of norepinephrine in the right ventricle (Table 13) was greater than that in the left ventricle at 8 weeks of myocardial infarction [36]. On the other hand, norepinephrine turnover did not change in the right ventricle (Table 13) but was enhanced in the left ventricle at 8 weeks of infarction [36]. These observations indicate differential alterations in the sympathetic nervous system activity of both the right and left ventricles of the infarcted animals. Considering the hypertrophic response of the heart to catecholamines as well as their adaptive and maladaptive effects on the signal transduction and subcellular mechanisms [10,30,31,32,36,46,54], it is suggested that the observed alterations in behavior of the right ventricle due to myocardial infarction may be a consequence of increased sympathetic activity as well as changes in both plasma levels and cardiac levels of catecholamines.

**Figure 1:**
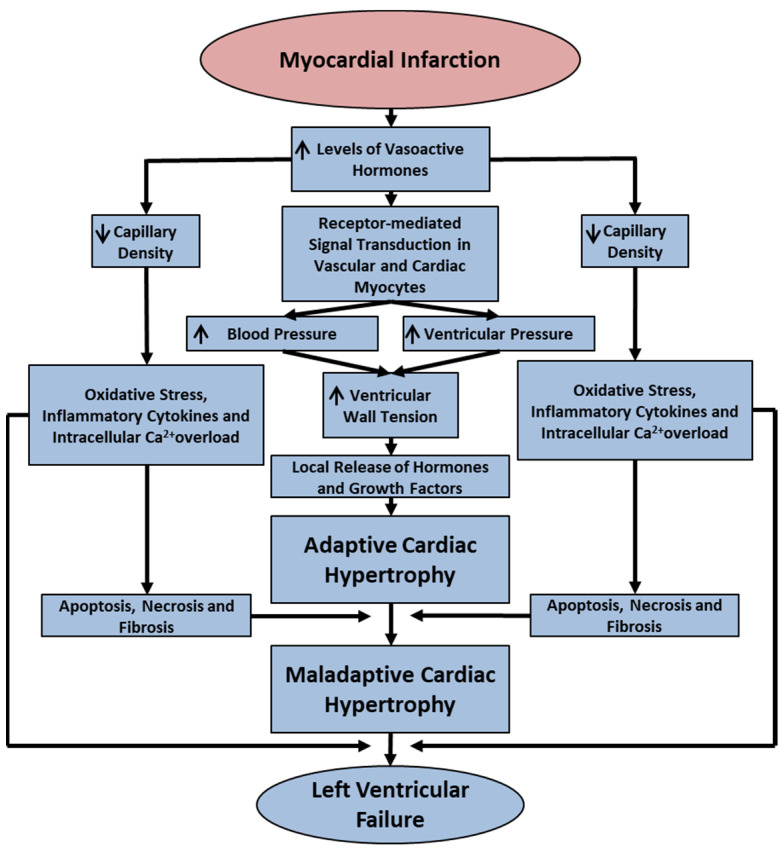
Schematic representation of some pathophysiological and cellular processes in the development of adaptive and maladaptive cardiac hypertrophy as well as left ventricular failure upon the induction of myocardial infarction. ↓—decrease; ↑—increase.

It is now well known that mechanisms for the development of cardiac hypertrophy and heart failure in different pathological situations are of complex nature and involve several factors such as vasoactive hormones, hemodynamic changes, various signal transduction pathways, metabolic alterations, oxidative stress, myocardial inflammation and intracellular Ca^2+^ overload [9,10,39,54,55,56,57]. Since myocardial infarction is the major cause of heart failure, some of the salient cellular and pathological processes for the occurrence of adaptive and maladaptive cardiac hypertrophy as well as heart failure are shown in Figure 1. It is also pointed and that while extensive experimental and clinical studies on heart failure over the past six decades have been carried out to understand the behavior of the left ventricle, relatively little information regarding the development of cardiac hypertrophy and failure in the right ventricle is available in the literature. Despite the fact that the most common cause of right ventricular dysfunction has been shown to be left ventricular failure [20,21], no specific intervention for the treatment of right ventricular failure has been developed. Thus, in the present article, we have not only put together the scattered information regarding the mechanisms of right ventricular adaptive and maladaptive hypertrophy under conditions of left heart failure due to myocardial infarction but have also identified molecular targets for the future development of improved therapy for right heart failure.

Although cardiac hypertrophy has been reported to occur in both the left ventricle and the right ventricle during the development of heart failure or maladaptive hypertrophy due to myocardial infarction [55,56,57], there are some similarities and differences in mechanisms for the occurrence of these phenotypes in left and right hearts. Cardiac hypertrophy due to myocardial infarction in the left ventricle has been shown to be due the both pressure and volume overload and is associated with an increase in myocyte length and diameter, whereas that in the right ventricle has been demonstrated to be due to pressure overload as well as an increase in myocyte length only [12,24,25,26,27,28,29]. It should also be noted that pressure overload and volume overload have been reported to produce eccentric and concentric types of hypertrophy in the left ventricle, respectively [10,15,16,17,18,19]. Furthermore, pressure overload on the right ventricle due to constriction of the pulmonary artery has been shown to induce adaptive hypertrophy initially, followed by cardiac dysfunction [12,13,14,58]. In fact, the behavior of right ventricular during the development of pulmonary hypertension [59] as well as in patients with chronic heart failure [60,61] is significantly influenced by sex differences. Furthermore, sex difference in the right ventricular structure and function have also been observed in heart failure patients with both reduced and preserved ejection fraction [62,63,64]. Particularly, it is noteworthy that there occurs a close relationship between the right ventricular volume and prognosis in patients with chronic heart failure [65]. Since females have better right ventricle performance, dysfunction of the right ventricle has been reported to be strong predictor of poor outcome of patients with pulmonary artery disease [59].

## 6. Concluding Remarks

In this article, we have reviewed the mechanisms of cardiac hypertrophy and remodeling in the right ventricle at different stages of left ventricular failure due to myocardial infarction. The occurrence of cardiac hypertrophy in the right ventricle was also evident from the observed changes in the myosin isozyme composition of the infarcted heart. Right ventricle hypertrophy was observed to be a direct or indirect consequence of different signal transduction pathways, including the activation of protein kinase C, phospholipase C and protein kinase A systems. Different investigators have demonstrated that the right ventricle function may be augmented during early periods of myocardial infarction, whereas cardiac dysfunction may occur in the hypertrophied right ventricle at late stages of heart failure. Since Ca^2+^ handling by the sarcoplasmic reticulum was enhanced, unlike the myofibrillar Ca^2+^-stimulated ATPase activity at early and moderate stages of heart failure due to myocardial infarction, it appears that the hypertrophied right ventricle may serve as a compensatory mechanism to support the function of the left ventricle at early and moderate stages of heart failure. Our observations also show that the Ca^2+^ uptake activity of the sarcoplasmic reticulum was depressed slightly (about 15%), whereas the myofibrillar Ca^2+^-stimulated ATPase activity was depressed significantly by about 18 to 20%, which seems to indicate that the right ventricle may be at the stage of maladaptive cardiac hypertrophy at 16 weeks after inducing myocardial infarction. It should be pointed out that after 16 weeks of myocardial infarction, the animals showed a severe stage of left ventricle failure. Thus, it is evident that the right ventricle exhibited adaptive cardiac hypertrophy and augmented cardiac function at early periods of infarction, whereas its function may have begun to be compromised due to the development of maladaptive cardiac hypertrophy at later periods of induced myocardial infarction.

**Figure 2:**
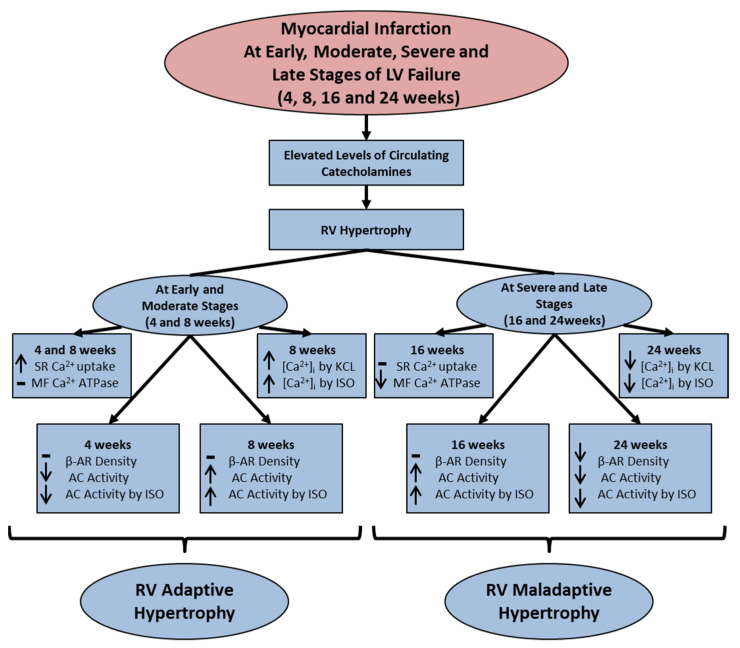
Graphical sketch of major changes in the right ventricle during the development of adaptive and maladaptive RV hypertrophy at 4 and 8 weeks (early and moderate stages of LV failure) as well as at 16 and 24 weeks (severe and late stages of LV failure) after inducing myocardial infarction in rats. LV, left ventricle; RV, right ventricle; −, no change; ↑—increase; ↓—decrease; SR, sarcoplasmic reticulum; MF, myofibril; KCL, potassium chloride; ISO, isoproterenol; β-AR, β-adrenoreceptor; AC, adenylyl cyclase.

At the moderate stage (8 weeks) of left ventricular failure due to myocardial infarction, the right ventricle showed an increase in the [Ca^2+^]_i_ due to the exposure of cardiomyocytes to a β-adrenoreceptor stimulant, isoproterenol, whereas such a response was depressed at late stages (24 weeks) of heart failure. The right ventricle at 8 weeks after infarction induction also showed an increase in adenylyl cyclase activity in the absence or presence of isoproterenol without any changes in the β-adrenoreceptor density. On the other hand, both β-adrenoreceptor density and adenylyl cyclase activity in the absence or presence of isoproterenol were depressed in the right ventricle at 24 weeks after inducing myocardial infarction. These observations indicate the upregulation and downregulation of β_1_-adrenoreceptor mechanisms at moderate and late stages of heart failure upon inducing myocardial infarction. Furthermore, the activation of adenylate cyclase by isoproterenol was not associated with any change in β_1_-adrenoreceptor density in the right ventricle at early, moderate or severe stages of heart failure due to myocardial infarction. Such isoproterenol-induced changes in adenylyl cyclase activity were observed to be due to an increase in the catalytic activity of the enzyme in the right ventricle, as increased activity of adenylyl cyclase was seen in the absence or presence of different agents at 8 and 16 weeks of infarction. The interaction of pertussis toxin with adenylyl cyclase revealed no change in ADP ribosylation or G_iα_-protein content in the right ventricle at 8 and 16 weeks of myocardial infarction. In addition, the depressed adenylyl cyclase activity in the absence or presence of isoproterenol at 4 weeks of myocardial infarction was probably due to increased G_iα_-protein content as well as ADP ribosylation. On the other hand, the interaction of cholera toxin with adenylyl cyclase showed an increase in ADP ribosylation and G_s_-protein content in the right ventricle at 8 and 16 weeks of infarction. These results indicate that the increased adenylyl cyclase activity in the right ventricle at different stages of heart failure may also be at the level of regulation of the enzyme by increased content as well as activity of G_s_ proteins. All the observed changes in the hypertrophic process, signal transduction pathway, subcellular mechanisms and β-adrenoreceptor pathway in the right ventricle at different stages of heart left ventricle failure due to myocardial infarction are shown graphically in Figure 2. These alterations in the right ventricle occurring during the development of adaptive as well as maladaptive cardiac hypertrophy seem to be a consequence of the elevated levels of circulating catecholamines due to changes in the sympathetic nervous system. However, extensive work needs to be carried out for establishing the role of the renin–angiotensin system in this regard.

## Figures and Tables

**Table 1:** General characteristics and hemodynamic parameters in control as well as early, moderate, severe and late stages of heart failure at 4, 8, 16 and 24 weeks after left coronary artery occlusion in rats.

Parameters	Control	Early Stage	Moderate Stage	Severe Stage	Late Stage
		(4 Weeks MI)	(8 Weeks MI)	(16 Weeks MI)	(24 Weeks MI)
**Scar wt** (g):					
	ND	0.361 ± 0.061	0.371 ± 0.082	0.351 ± 0.061	0.350 ± 0.052 *
**Viable Left Ventricle wt** (g):					
	0.805 ± 0.086	0.794 ± 0.092	1.273 ± 0.172 *	1.475 ± 0.103 *	1.548 ± 0.149 *
**Right Ventricle wt** (g):					
	0.235 ± 0.033	0.331 ± 0.027 *	0.392 ± 0.041 *	0.427 ± 0.047 *	0.546 ± 0.026 *
**Ascites** (mL):					
	ND	3.4 ± 1.1 *	6.2 ± 1.9 *	12.0 ± 0.2 *	13.2 ± 1.4 *
**Left Ventricle Systolic Pressure** (mmHg):					
	125 ± 5.2	131 ± 6.1	121 ± 2.8	93 ± 4.1 *	84 ± 3.9 *
**Left Ventricle Diastolic Pressure** (mmHg):					
	2.1 ± 0.1	11.5 ± 2.2 *	12.0 ± 1.3 *	15 ± 0.7 *	20 ± 4.3 *
**Left Ventricle + dP/dt** (mmHg/s):					
	5986 ± 197	4691 ± 172 *	4691 ± 172 *	3645 ± 330 *	3608 ± 218 *
**Left Ventricle − dP/dt** (mmHg/s):					
	5184 ± 272	3507 ± 197 *	382 ± 214 *	3152 ± 284 *	3314 ± 168 *

The data are based on the information in our papers “Sethi et al., 1998 [31] and Sethi et al., 2006 [32]”. Each value is a mean ± SE of 10 experiments. MI, myocardial infarction; viable left ventricle weight is without scar; ±dP/dt, rate of contraction; −dP/dt, rate of relaxation; ND, not detectable. * *p* < 0.05 vs. respective control value.

**Table 2:** Protein kinase C (PKC) activities in control as well as the right ventricle at 2, 4 and 8 weeks after left coronary artery occlusion in rats.

Parameters	Pre-Failure Stage		Early Failure Stage		Moderate Failure Stage	
	Sham	(2 Weeks MI)	Sham	(4 Weeks MI)	Sham	(8 Weeks MI)
**Right ventricle wt** (g)						
	163 ± 5.2	216 ± 17.2 *	172 ± 12.6	306 ± 33.1 *	278 ± 17.1	600 ± 19.8 *
**Ca^2+^-dependent PKC activity** (pmol ^32^P/min/mg protein)						
	126 ± 4.7	156 ± 15.3 *	98.7 ± 6.8	132 ± 13.4 *	93.5 ± 6.4	184 ± 14 *
**Ca^2+^-independent PKC activity** (pmol ^32^P/min/mg protein)						
	118 ± 4.9	146 ± 8.9 *	106 ± 2.6	137 ± 4.7 *	70.5 ± 5.2	238 ± 18.5 *

Values are mean ± SE of six experiments. Both Ca^2+^-dependent and Ca^2+^-independent PKC activities were measured in homogenate. The data are based on the information in our paper, Wang et al., 2003 [34]. * *p* < 0.5 vs. respective control; MI, myocardial infarction.

**Table 3:** Relative contents of protein kinase C (PKC) isozymes (α, β, Ɛ, ꞔ), as well as protein kinase A (PKA), in the right ventricle at 8 weeks of myocardial infarction (MI) in rats.

Parameters	Sham	Moderate Failure Stage (8 Weeks MI)
**A. Protein kinase C** (% of control):		
PKC-α	100	138 ± 11 *
PKC-β	100	145 ± 16 *
PKC-ℇ	100	126 ± 5 *
PKC-ꞔ	100	108 ± 3
**B. Protein kinase A**		
PKA content (% of control):	100	
PKA activity (pmol ^32^P/min/mg protein):	3600 ± 356	3580 ± 375

Values are mean ± SE of six experiments. PKC isozymes as well as PKA contents and activity were measured in homogenate. The data are based on the information in our paper, Wang et al., 2003 [34]. * *p* < 0.05 vs. sham.

**Table 4:** Phospholipase C (PLC) activity and kinetic parameters in sarcolemma from the right ventricle at 8 weeks (moderate stage) and 16 weeks (severe stage) of heart failure due to myocardial infarction (MI).

**Parameters**	**Moderate Failure Stage**		**Severe Failure Stage**	
	**Control**	**(8 Weeks MI)**	**Control**	**(16 Weeks MI)**
**A. PLC activity** (nmol IP_3_/min/mg protein):	4.35 ± 0.64	4.23 ± 0.37	5.42 ± 0.60	3.7 ± 0.58 *
**B. Kinetic parameters**				
V_max_ (nmol IP_3_/min/mg protein):	35.5 ± 4.2	36.9 ± 5.4	37.7 ± 3.1	40.1 ± 7.0
K_m_ (μM PIP_2_):	123 ± 11	138 ± 6	118 ± 3	193 ± 7 *

Values are mean ± SE of three to six experiments. PLC activity (A) was measured by employing 20 μM phosphatidylinositol 4,5-bisphosphate (PIP_2_), whereas kinetic parameters were determined by employing 5 to 200 μM PIP_2_ in the incubation medium. V_max_ and K_m_ values were determined from Lineweaver–Burk plots of PIP_2_ hydrolysis to produce inositol-1,4,5-triphosphate (IP_3_). The data are based on the information in our paper, Meij et al., 1997 [35]. * *p* < 0.05 vs. control.

**Table 5:** Sarcoplasmic reticulum Ca^2+^ uptake and kinetic parameters in the right ventricle from the control and different stages of heart failure at 4, 8 and 16 weeks after inducing myocardial infarction (MI) in rats.

Parameters	Control	Early Failure	Moderate Failure	Severe Failure
		(4 Weeks MI)	(8 Weeks MI)	(8 Weeks MI)
**A. Calcium uptake** (nmol Ca^2+^/min/mg protein)	29 ± 1.9	42 ± 1.6 *	35 ± 1.3*	25 ± 0.9
**B. Kinetic parameters**				
V_max_ (nmol/min/mg protein):	30 ± 1.5	45 ± 0.9 *	37 ± 1.3 *	25 ± 1.2
K_m_ (μM Ca^2+^):	0.61 ± 0.04	0.52 ± 0.06	0.52 ± 0.09	0.58 ± 0.05

Values are mean ± SE of four to six experiments. Calcium uptake (A) was measured by using 10 μM Ca^2+^, whereas kinetic parameters were determined by employing 0.1 μM to 10 μM Ca^2+^ in the incubation medium. V_max_ and K_m_ values were determined by Lineweaver–Burk plot analysis. The data are based on information in our paper, Afzal et al., 1992 [37]. * *p* < 0.05 vs. respective control.

**Table 6:** Myofibrillar Ca^2+^-stimulated ATPase as well as myosin protein and gene expression in the right ventricle from control and different stages of heart failure at 4, 8 and 16 weeks after inducing myocardial infarction (MI) in rats.

Parameters	Control	Early Failure	Moderate Failure	Severe Failure
		(4 Weeks MI)	(8 Weeks MI)	(16 Weeks MI)
**A. Myofibrillar Ca^2+^-stimulated ATPase activity** (μmolPi/h/mg protein):				
	8.9 ± 035	8.1 ± 0.33	8.0 ± 0.38	7.3 ± 0.23 *
**B. Myosin protein and isozyme expression at 8 weeks MI**				
Protein content (% of control):			95 ± 2.4	
Myosin isozymes (% of total)				
Decrease in myosin α:			57 ± 5 *	
Increase in myosin β:			43 ± 4 *	
**C. Myosin mRNA expression** (at 8 weeks MI)				
Decrease in myosin α mRNA (% of control)			45 ± 5 *	
Increase in myosin β mRNA (% of control)			40 ± 5 *	

Values are mean ± SE of six experiments. The data are based on the information in our paper, Wang et al., 2002 [42]. Values for myosin mRNA expression were determined as myosin α mRNA/GAPDH mRNA and myosin β mRNA/GAPDH mRNA ratios. Myofibrillar Mg^2+^–ATPase activity varied between 2.8 and 3.7 μmolPi/h/mg protein, and there was no statistical difference between control and experimental animals. * *p* < 0.05 vs. control.

**Table 7:** Isoproterenol-induced increase in intracellular Ca^2+^ concentration [Ca^2+^]_I_ in KCL-depolarized myocytes from the right ventricle at moderate (8 weeks) and late (24 weeks) stages of heart failure due to myocardial infarction (MI) in rats.

Parameters	Moderate Failure Stage		Late Failure Stage	
	Control	(8 Weeks MI)	Control	(24 Weeks MI)
**A. Basal**				
**[Ca^2+^]_i_** (nmol/L):	83 ± 7.1	82 ± 5.2	82 ± 6.4	87 ± 5.3
**B. KCL-induced increase**				
**[Ca^2+^]_i_** (nmol/L):	52 ± 3.4	78 ± 4.9 *	56 ± 2.8	38 ± 1.7 *
**C. Isoproterenol-induced increase**				
**[Ca^2+^]_i_** (nmol/L):	33 ± 4.8	48 ± 5.6 *	36 ± 3.1	24 ± 2.2 *

Values are mean ± SE from four hearts in each group. The concentration of KCL was 30 nmol/L, whereas the concentration of isoproterenol was 100 μmol/L. The data are based on the information in our paper, Sethi et al., 2006 [32]. * *p* < 0.05 vs. respective control.

**Table 8:** Maximal binding (B_max_) and dissociation constant (K_d_) for β-adrenoreceptors as well as basal and isoproterenol-stimulated adenylyl cyclase activities in the right ventricle at moderate (8 weeks) and late (24 weeks) stages of heart failure due to myocardial infarction (MI) in rats.

Parameters	Moderate Failure Stage		Late Failure Stage	
	Control	(8 Weeks MI)	Control	(24 Weeks MI)
**A. β-adrenoreceptors**				
B_max_ (f mol/mg protein)	54 ± 3.7	58 ± 4.1	51 ± 2.9	36 ± 2.7 *
K_d_ (p mol)	49 ± 6.4	55 ± 5.2	54 ± 6.0	49 ± 4.6
**B. Adenylyl cyclase activity** (pmol cyclic AMP/10 min/mg protein)				
Basal:	121 ± 8.9	152 ± 7.6 *	123 ± 6.8	65 ± 4.2 *
Isoproterenol:	327 ± 18.5	486 ± 29.7 *	290 ± 18.5	113 ± 8.6 *

Values are mean ± SE of four hearts in each group. (^125^I)-iodocyanopindolol was used for β-adrenoreceptor binding. Adenylyl cyclase activity was determined in the absence (basal) or presence of 10 μM isoproterenol in a reaction medium containing 10 μmol 5′-guanylyl imidodiphosphate/L. The data are based on the information in our paper, Sethi et al., 2006 [32]. * *p* < 0.05 vs. respective control.

**Table 9:** Status of β_1_-adrenreceptors and adenylyl cyclase activity in the presence of isoproterenol (100 μM) in the right ventricle from the control as well as different stages of heart failure at 4, 8 and 16 weeks of myocardial infarction (MI) in rats.

Parameters	Control	Early Failure Stage (4 Week MI)	Moderate Failure Stage (8 Weeks MI)	Severe Failure Stage (16 Weeks MI)
**A. β_1_-Adrenoreceptors**				
B_max_ (f mol/mg protein):	24 ± 2.3	29 ± 3.1	28 ± 3.1	27 ± 3.2
K_d_ (pM):	48 ± 3.3	49 ± 3.4	49 ± 4.0	49 ± 4.1
**B. β_2_-Adrenoreceptors**				
B_max_ (f mol/mg protein):	8 ± 1.9	7 ± 1.1	9 ± 2.0	10 ± 1.8
K_d_ (pM):	13 ± 1.2	14 ± 1.0	11 ± 1.3	12 ± 1.4
**C. Adenylyl cyclase activity** (pmol cyclic AMP/10 min/mg protein)				
Isoproterenol	402 ± 35	258 ± 21 *	640 ± 42 *	648 ± 45 *

Values are mean ± SE of four to six animals. Both maximal binding (B_max_) and dissociation constants (K_d_) were calculated from the Scatchard plot analysis. (^125^I) iodocyanopindolol binding was obtained by employing CGP-20712A for β_1_-adrenoreceptors and ICI-118551 for β_2_-adrenoreceptors. Determination of adenylyl cyclase in the presence of isoproterenol was carried out by using 10 μM Gpp(NH)p in the assay medium. The data are based on information in our paper, Sethi et al., 1997 [46]. * *p* < 0.05 vs respective control.

**Table 10:** Adenylyl cyclase activity in the absence (basal) and presence of NaF, forskolin and Gpp (NH)p in the right ventricle from the control as well as at early (4 weeks), moderate (8 weeks) and severe (16 weeks) stages of heart failure due to myocardial infarction (MI) in rats.

Parameters	Control	Early Failure Stage (4 Weeks MI)	Moderate Failure Stage (8 Weeks MI)	Severe Failure Stage (16 Weeks MI)
Adenylyl cyclase activity (pmol cyclic AMP/10 min/mg protein):				
Basal	114 ± 15	66 ± 8 *	156 ± 8 *	163 ± 10*
NaF (5 mM)	570 ± 54	244 ± 36 *	936 ± 76 *	720 ± 31 *
Foskolin (100 μM)	353 ± 23	183 ± 22 *	624 ± 45 *	484 ± 12 *
Gpp (NH)p (30 μM)	300 ± 16	171 ± 12 *	445 ± 21 *	455 ± 9 *

Values are mean ± SE of four to six experiments. The data are based on the information in our paper, Sethi et al., 1997 [46]. * *p* < 0.05 vs. control.

**Table 11:** Adenylyl cyclase activity and ADP ribosylation in the absence and presence of pertussis toxin (5 μg/mL) as well as G_iα_-protein content in the right ventricle from the control as well as at early (4 weeks), moderate (8 weeks) and severe (16 weeks) stages of heart failure due to myocardial infarction (MI) in rats.

Parameters	Control	Early Failure Stage (4 Weeks MI)	Moderate Failure Stage (8 Weeks MI)	Severe Failure Stage (16 Weeks MI)
**A. Adenylyl cyclase activity** (pmol cyclic AMP/10 min/mg protein):				
Basal	148 ± 9.5	120 ± 8.6 *	184 ± 11.2 *	186 ± 10.7 *
Pertussis toxin	214 ± 12.9	278 ± 15.6 *	218 ± 12.8	206 ± 10.9
**B. ADP ribosylation**				
Pertussis toxin catalyzed (% control):	100	162 ± 12 *	111 ± 10	107 ± 7
**C. G-protein content**				
G_iα_ protein (% control)	100	172 ± 28 *	94 ± 9	107 ± 7

Values are mean ± SE of five experiments. ADP ribosylation and G_iα_-protein content were measured at the 42 KD band. The data are based on information in our paper, Sethi et al., 1998 [31]. * *p* < 0.05 vs. respective control.

**Table 12:** Adenylyl cyclase activity and ADP ribosylation in the absence and presence of cholera toxin (20 μg/mL) as well as G_s_-protein content in right ventricle from the control as well as at early (4 weeks), moderate (8 weeks) and severe (16 weeks) stages of heart failure due to myocardial infarction (MI) in rats.

Parameters	Control	Early Failure Stage (4 Weeks MI)	Moderate Failure Stage (8 Weeks MI)	Severe Failure Stage (16 Weeks MI)
**A. Adenylyl cyclase activity** (pmol/cyclic AMP/10 min/mg protein):				
Basal	148 ± 7.6	115 ± 5.8 *	208 ± 17.9 *	200 ± 14.5 *
Cholera Toxin	292 ± 15.8	240 ± 8.9 *	486 ± 23.7 *	478 ± 26.2 *
**B. ADP ribosylation**				
Cholera toxin catalyzed at				
45 KD (% control)	100	98 ± 8	148 ± 12 *	137 ± 12 *
52 KD (% control)	100	97 ± 10	144 ± 10 *	129 ± 12
**C. G-protein content**				
G_s_ protein 45 KD (% of control)	100	105 ± 10	300 ± 14 *	213 ± 22 *
G_s_ protein 52 KD (% of control)	100	101 ± 9	208 ± 10 *	152 ± 10 *

Values are mean ± SE of five experiments. ADP ribosylation and G_s_-protein contents were measured at 45 KD and 52 KD bands. The data are based on information in our paper, Sethi et al., 1998 [31]. * *p* < 0.05 vs. respective control.

**Table 13:** Plasma and right ventricle catecholamines as well as norepinephrine turnover in the right ventricle in early (4 weeks MI) and moderate (8 weeks MI) stages of heart failure due to myocardial infarction (MI) in rats.

Parameters	Control	Early Failure Stage (4 Weeks MI)	Moderate Failure Stage (8 Weeks MI)
**Plasma norepinephrine** (pg/mL):	920 ± 160	1076 ± 143	1820 ± 208 *
**Plasma epinephrine** (pg/mL):	846 ± 209	1868 ± 165 *	1680 ± 248 *
**Right ventricle norepinephrine** (ng/g)	224 ± 12	208 ± 17	265 ± 20 *
**Right ventricle Epinephrine** (ng/g)	18 ± 3.2	38 ± 2.6 *	42 ± 3.8 *
**Norepinephrine Turnover** (Kh^−1^)	0.12 ± 0.008	ND	0.13 ± 0.01
**Initial radioactivity** (DPM)	3940 ± 180	ND	7264 ± 235 *

Values are mean ± SE of four to eight experiments. The data are based on information in our paper, Ganguly et al., 1997 [36]. * *p* < 0.05 vs. respective control. ND, not determined.

## Data Availability

Not applicable.
